# Advances in understanding telomerase assembly

**DOI:** 10.1042/BST20230269

**Published:** 2023-12-18

**Authors:** Basma M. Klump, Jens C. Schmidt

**Affiliations:** 1 Institute for Quantitative Health Sciences and Engineering, Michigan State University, East Lansing, MI, U.S.A.; 2 College of Osteopathic Medicine, Michigan State University, East Lansing, MI, U.S.A.; 3 Cell and Molecular Biology Graduate Program, College of Natural Sciences, Michigan State University, East Lansing, MI, U.S.A.; 4 Department of Obstetrics, Gynecology, and Reproductive Biology, Michigan State University, East Lansing, MI, U.S.A.

**Keywords:** Cajal bodies, ribonucleoproteins, telomerase, telomeres

## Abstract

Telomerase is a complex ribonucleoprotein scaffolded by the telomerase RNA (TR). Telomere lengthening by telomerase is essential to maintain the proliferative potential of stem cells and germ cells, and telomerase is inappropriately activated in the majority of cancers. Assembly of TR with its 12 protein co-factors and the maturation of the 5′- and 3′-ends of TR have been the focus of intense research efforts over the past two decades. High-resolution Cryo-EM structures of human telomerase, high-throughput sequencing of the 3′ end of TR, and live cell imaging of various telomerase components have significantly advanced our understanding of the molecular mechanisms that govern telomerase biogenesis, yet many important questions remain unaddressed. In this review, we will summarize these recent advances and highlight the remaining key questions with the ultimate goal of targeting telomerase assembly to suppress telomere maintenance in cancer cells or to promote telomerase activity in patients affected by telomere shortening disorders.

## Introduction

**Figure 1.**
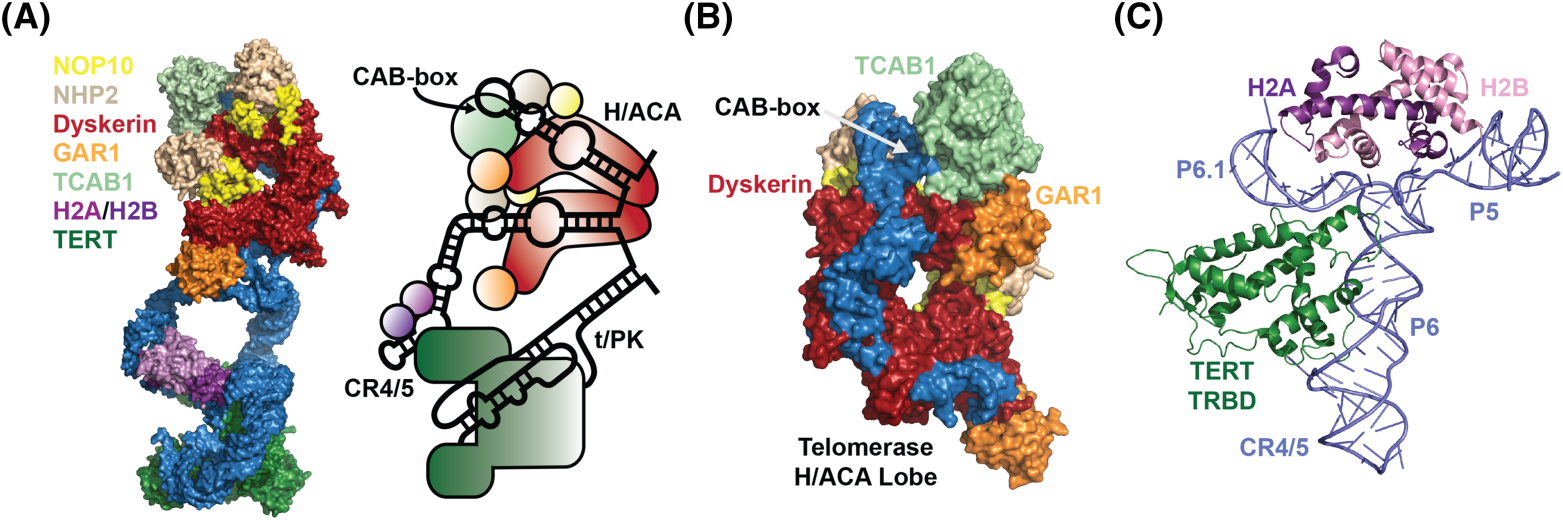
Structure and composition of the human telomerase RNP. (**A**) Overall structure and organization of the telomerase RNA and the 12 associated protein components of telomerase (adapted from Ghanim et al. [4]). (**B**) Structure of the H/ACA lobe of the human telomerase RNP highlighting the interface between TCAB1, dyskerin, GAR1, and TR (based on PDB: 7TRC, Liu et al. [7]). (**C**) Structure of the CR4/5 region of TR bound by the histone H2A/H2B dimer and the TERT TRBD domain (based on PDB: 7BG9, Ghanim et al. [4]).

The telomerase ribonucleoprotein (RNP) elongates telomeres in human stem cells and germ cells by adding TTAGGG repeats to the chromosome end, which maintains their proliferative potential [1]. In the absence of telomerase, telomeres shorten every cell division due to the end replication problem, resulting in cell cycle arrest or apoptosis [2]. The human telomerase RNP is composed of the telomerase RNA (TR), telomerase reverse transcriptase (TERT), telomerase Cajal body protein 1 (TCAB1), along with two copies of the H/ACA complex containing dyskerin (DKC1), GAR1, Non-Histone Protein 2 (NHP2) and Nucleolar Protein 10 (NOP10) (Figure 1A) [3–5]. Furthermore, multiple independent cryogenic electron microscopy (Cryo-EM) structures have recently identified an H2A/H2B histone dimer as an additional component of the human telomerase RNP [4,6,7]. Mutations in all telomerase RNP factors, with the exception of GAR1, have been identified in a variety of multisystem diseases known as the telomere syndromes [8]. Telomere syndromes, including dyskeratosis congenita, idiopathic pulmonary fibrosis, Hoyeraal–Hreiderson syndrome, and aplastic anemia, are a set of pre-mature ageing diseases defined by very short telomeres in rapidly dividing stem cell populations such as the bone marrow or lung epithelium [8,9]. While a reduction in telomerase activity is associated with telomere syndromes, telomerase expression is required for telomere maintenance and proliferation of >85% of human cancers [10]. Defining the molecular mechanisms of telomerase biogenesis could therefore lead to targeted approaches to increase telomerase activity in patients affected by telomere syndromes, or to inhibit telomerase activity in malignant neoplasms. In this review, we will summarize recent advances in our understanding of telomerase biogenesis in human cells.

## TR biogenesis

The telomerase RNA (TR) is a 451-nucleotide non-coding RNA, that is transcribed by RNA polymerase II [11]. TR contains two functional domains: the catalytic lobe, which includes the template, pseudo-knot (t/PK) and conserved regions 4 and 5 (CR4/5) bound by TERT and H2A/H2B, and the H/ACA lobe, which associated with two H/ACA complexes and TCAB1 [3–5]. The two lobes are connected by two flexible regions of TR (Figure 1A) [4,5].

**Figure 2.**
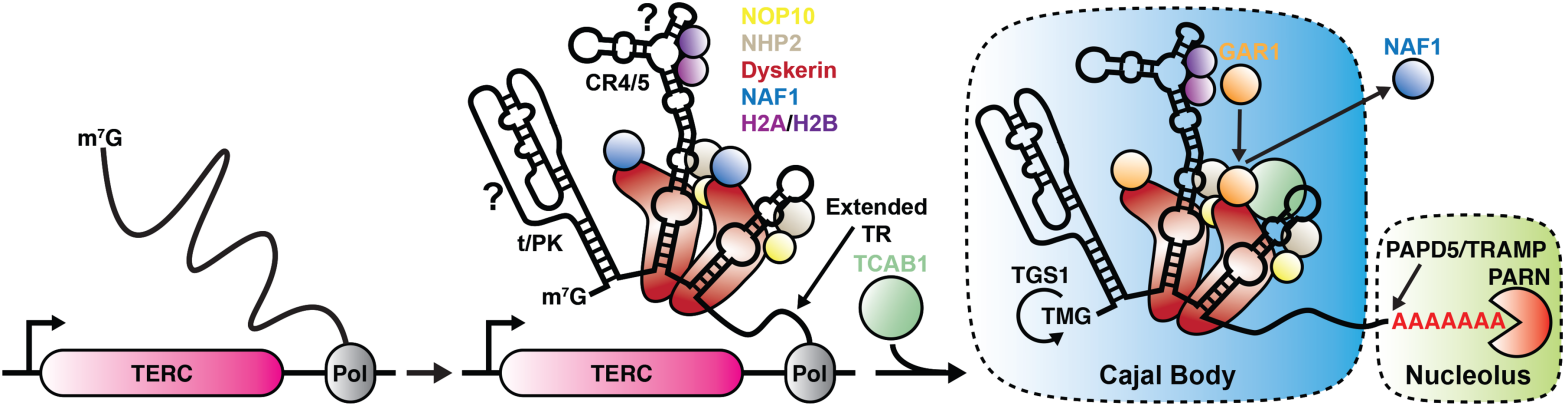
Model of human telomerase RNA biogenesis. The H/ACA region of m7G-modified TR folds co-transcriptionally and binds to the dyskerin–NHP2–NOP10–NAF1 complex. It is unknown whether t/PK and CR4/5 folding alongside H2A/H2B binding also occur during transcription. Post-transcriptional modifications of TR include TMG-cap formation by TGS1 in Cajal bodies, 3′-polyadenylation by PADP5, and exonucleolytic trimming by PARN both of which are enriched in nucleoli. In addition, TR binds TCAB1, and NAF1 is exchanged for GAR1.

TR shares the H/ACA region with small Cajal body RNAs (scaRNAs) and small nucleolar RNAs (snoRNAs), which serve as guides for the pseudouridine synthase activity of dyskerin [12,13]. Similar to snoRNAs and scaRNAs, dyskerin is thought to associate with TR co-transcriptionally [14]. In addition, like other Pol II transcripts, TR is 7-methyl guanosine (m^7^G) capped during transcription [15]. Importantly, TR transcription does not terminate after 451 nucleotides have been synthesized, but typically proceeds past this point leading to the production of extended TR transcripts (Figure 2) [15–17]. Interestingly, an extended form of TR has been shown to form a triple helix with the H/ACA domain of TR, which competes with dyskerin binding and leads to TR degradation [18]. After transcription, the 5′- and 3′-ends of TR are both further modified. The poly-A polymerase PAPD5, as part of the TRAMP complex, adds a poly-A tail to the 3′-end of TR (Figure 2) [15–17]. Following polyadenylation, the poly-A binding RNase factor (PABPN1) and poly(A)-specific ribonuclease (PARN) degrade the poly-adenylated 3′-end and additional nucleotides added during transcription of TR leading to the formation of the mature 451-nucleotide-long telomerase (Figure 2) [15–17]. The boundary of exonucleolytic degradation by PARN is established by dyskerin, which directly associates with the terminal nucleotides of TR, likely forming a physical barrier preventing further nuclease action [4]. In the absence of dyskerin or PARN, TR becomes vulnerable to degradation by the exosome, which recognizes the poly-adenylated end of TR [15,19,20]. For this reason, mutations in PARN and dyskerin are associated with telomere syndromes due to a reduction in TR levels [17,21,22]. Importantly, preventing polyadenylation of TR by depleting or inhibiting PAPD5, increases TR stability and is a promising approach to treat telomere syndromes caused by mutations in factors affecting TR accumulation [21,23].

In addition to the 3′-end binding factors that promote TR maturation, TR transcripts also receive a trimethyguanosine (TMG) cap at the 5′-end which contributes to its accumulation and maturation [13]. Trimethyguanosine synthase 1 (TGS1) localizes to Cajal bodies (CBs) and mediates TMG cap formation on TR (Figure 2) [13,24]. Interestingly, when TGS1 activity is reduced, TR levels increase, telomerase activity is elevated, and telomeres elongate [25,26]. Together these observations demonstrate that TMG cap formation is an important regulatory step in TR maturation. Importantly, 5′- and 3′-end modification appear to be co-ordinated since m^7^G-cap binding proteins, but not the TMG cap, can both recruit the exosome via the NEXT complex or promote polyadenylation and PARN mediated maturation of TR by recruiting the TRAMP complex [15].

## Telomerase RNP components and their function

Recent Cryo-EM structures have precisely defined the composition and organization of the catalytic and H/ACA lobes of the telomerase RNP [3–5]. In the H/ACA lobe, dyskerin acts as an anchor for the co-factors of the H/ACA complex (Figure 1B). Recent findings suggest that dyskerin pre-assembles with NOP10, NHP2, NAF1, and SHQ1 prior to encountering TR [27]. In addition, dyskerin is SUMOylated, which directs its nucleolar localization and its interaction with GAR1 and TR [28]. SHQ1 promotes proper dyskerin folding by binding to its RNA-binding interface and preventing pre-mature association of dyskerin with RNA [29,30]. To allow binding of the dyskerin–NHP2–NOP10–NAF1 complex to TR, the AAA+ ATPases reptin and pontin remove SHQ1 from dyskerin [31]. In addition, NAF1 has been shown to interact with the c-terminal disordered domain of RNA polymerase II, which might contribute to the co-transcriptional association of the H/ACA complex with nascent TR (Figure 2) [32]. Once the H/ACA complex is bound to TR, NAF1 is replaced with GAR1 [14]. Recent findings suggest that NAF1 and GAR1 can co-occur in the same complex which might reflect their binding to distinct stem loops in snoRNPs or scaRNPs [27]. Functionally, the H/ACA complex is critical for stabilizing TR, by protecting it against exonucleolytic degradation. Mutations in dyskerin, NHP2, NAF1, and NOP10 all lead to reduction in TR levels and are associated with short telomeres [8,33].

The final component of the H/ACA lobe of telomerase is TCAB1, which binds to the Cajal body (CAB) box in the terminal hairpin of TR and other scaRNAs (Figure 1B) [13,34]. Unlike the components of the H/ACA complex, TCAB1 is not required for TR stability [34,35]. TR levels were shown to be unchanged in the absence of TCAB1 [35–38], or potentially slightly elevated [39]. Mutations in TCAB1 or the CAB-box in TR lead to telomere shortening in dyskeratosis congenita and Hoyeraal-Hreidarsson syndrome patients [35,40,41]. Interestingly, the disease-associated mutations found in TCAB1 are located in its WD40 domain and result in the binding of TCAB1 to the TRiC chaperonin, indicating that the mutations prevent proper TCAB1 folding [37]. Initial publications suggested that TCAB1 contributes to telomerase recruitment to telomeres [34,42]. A more recent study implicated TCAB1 in proper folding of the CR4/5 region of TR, which was proposed to reduce the interaction of TR with TERT and in turn the catalytic activity of telomerase [36]. The CR4/5 region is not part of the H/ACA lobe and is located a long distance away from TCAB1 in the context of the assembled telomerase RNP (Figure 1). The mechanism by which TCAB1 could regulate CR4/5 folding therefore remains to be determined. Rather than TCAB1 playing a role in TR folding and telomerase catalysis, our recent work suggests that TCAB1 is not required for telomerase activity, but instead promotes telomerase assembly in human cancer cells [39].

The core of the catalytic lobe of the telomerase RNP is formed by t/PK region of TR [43,44]. In addition, the catalytic lobe contains the CR4/5 region of TR, which emanates from the first stem of the H/ACA lobe and binds to the telomerase RNA-binding domain (TRBD) of TERT which is essential for telomerase assembly and activity (Figure 1A,C) [45]. Interestingly, a recent pre-print applied dimethyl sulfate mutational profiling to demonstrated that CR4/5 can adopt two distinct conformations [46]. The non-canonical conformation is less abundant (∼15%) than the canonical CR4/5 fold and fails to assemble with TERT to form an active telomerase RNP. CR4/5 also associates with the histone H2A/H2B dimer, which is cradled by the P5 and P6.1 stems of TR (Figure 1C) [4]. Since the H2A/H2B dimer appears to specifically recognize the surface formed by the P5 and P6.1 stems of the CR4/5 three-way junction, it is tempting to speculate that it stabilizes this conformation to promote proper folding of this critical region of TR. The association of TERT with TR has been shown to be an inefficient process. Human cancer cells can contain TERT that is not associated with TR and vice versa [47]. In contrast, TERT expression in human embryonic stem cells is sufficiently high to saturate all TR molecules, making TR the limiting factor for telomerase assembly and hence activity [48].

## Sub-cellular localization and order of telomerase assembly

While the molecular steps required for TR maturation and the molecular interactions within the telomerase RNP are well defined, the sub-cellular locations where specific maturation and assembly steps occur remain poorly understood. Cajal bodies and nucleoli, phase-separated nuclear organelles critical for the maturation of small nuclear RNAs (snRNAs) and ribosomal RNAs, respectively, have been implicated in telomerase assembly. Because TR is a box H/ACA scaRNA, it localizes with Cajal bodies [13]. It is unknown whether the TR locus, like other scaRNA loci, directly associates with Cajal bodies [49]. Under specific circumstances, for example when TCAB1 is knocked-out or the CAB-box is mutated, TR is enriched in nucleoli localizing similarly to snoRNAs [34,35,39,42].

The first steps of telomerase assembly, m^7^G-capping and association with the dyskerin–NHP2–NOP10–NAF1 complex likely occur co-transcriptionally (Figure 2). However, it is worth noting that co-transcriptional association of the H/ACA complex has only been demonstrated for the E3 snoRNA, but not TR [14]. Co-transcriptional binding of the H/ACA complex to TR suggests that the box H and ACA and the stem loops flanking them are rapidly formed during TR synthesis (Figure 2). The next step in H/ACA complex maturation is the replacement of NAF1 with GAR1. Since NAF1 is not enriched in Cajal bodies and is excluded from nucleoli [14], it must be replaced by GAR1 either prior to or immediately upon association of TR with Cajal bodies. Importantly, TCAB1 is required for TR localization to CBs and therefore must associate with TR prior to its recruitment. The Cryo-EM structure of the telomerase RNP revealed that TCAB1, in addition to binding to the CAB-box of TR, also interacts with dyskerin and GAR1 (Figure 1B) [4]. It is therefore possible that TCAB1 and GAR1 binding to TR are coordinated. Furthermore, since NAF1 and GAR1 bind to the same surface of dyskerin and NAF1 is a significantly larger protein than GAR1 (494 and 217 amino acids, respectively), NAF1 and TCAB1 binding to TR could be mutually exclusive due to steric hindrance. A final puzzling observation is that most of the TCAB1 protein in the cell localizes to the cytoplasm, rather than the nucleus, but TCAB1 lacks a clear nuclear important signal [35,39]. This suggests that TCAB1 might require assembly with other factors, such as scaRNPs, for nuclear import. It is possible that TR is transiently exported to the cytoplasm, like budding yeast TLC1 [50], to assemble with TCAB1 in human cells. In total, many open questions remain regarding the sequence and location of the assembly of the H/ACA complex of the telomerase RNP.

The sub-cellular location of TR maturation has not been directly determined, but can be inferred from localization of the key processing enzymes involved in 5′ and 3′ modification of TR. The TRAMP complex, which polyadenylates the 3′-end of TR, is enriched in the nucleolus [51]. Consistent with polyadenylation of TR in nucleoli, extended TR transcripts have been shown to localize to these organelles [16]. The NEXT complex which contributes to exonucleolytic degradation of poly-adenylated TR is found in the nucleoplasm and is excluded from the nucleolus [51]. The exclusion of factors that mediate exosomal degradation of TR from nucleoli could provide an explanation for the increase in TR levels, when TR accumulates in nucleoli in the absence of TCAB1 or TGS1 [25,39]. PARN localizes to both nucleoli and Cajal bodies, suggesting that the final maturation of the 3′ end of TR may occur in either or both of these compartments (Figure 2) [52]. Cajal bodies are also thought to be the site of TMG cap formation on TR. TGS1 is enriched in CBs, and like TCAB1, when TGS1 is depleted, TR localizes to nucleoli increasing its levels in human cancer cells [25]. Depletion of TGS1 is also associated with an increase in cytoplasmic TR levels, consistent with TMG-cap formation preventing m^7^G-cap mediated export of TR [25]. Cytoplasmic localization of TR was also observed when the 3′-end of TR was destabilized by PARN or dyskerin knockdown [19]. Whether this cytoplasmic accumulation of TR is part of the telomerase biogenesis process or a consequence of aberrant TR processing is unclear. Interestingly, CBs are not required for telomerase activity or telomere maintenance [36,53]. This suggests that, while some steps of telomerase biogenesis might occur in CBs, they can take place with similar efficiency when CBs are not present in human cells. For scaRNAs, that are involved in the modification of snRNAs, localization to CBs might be functionally important. However, since TR does not serve as a guide for dyskerin mediated pseudo-uridylation, its localization to CBs might not play a role in telomerase RNP function.

**Figure 3.**
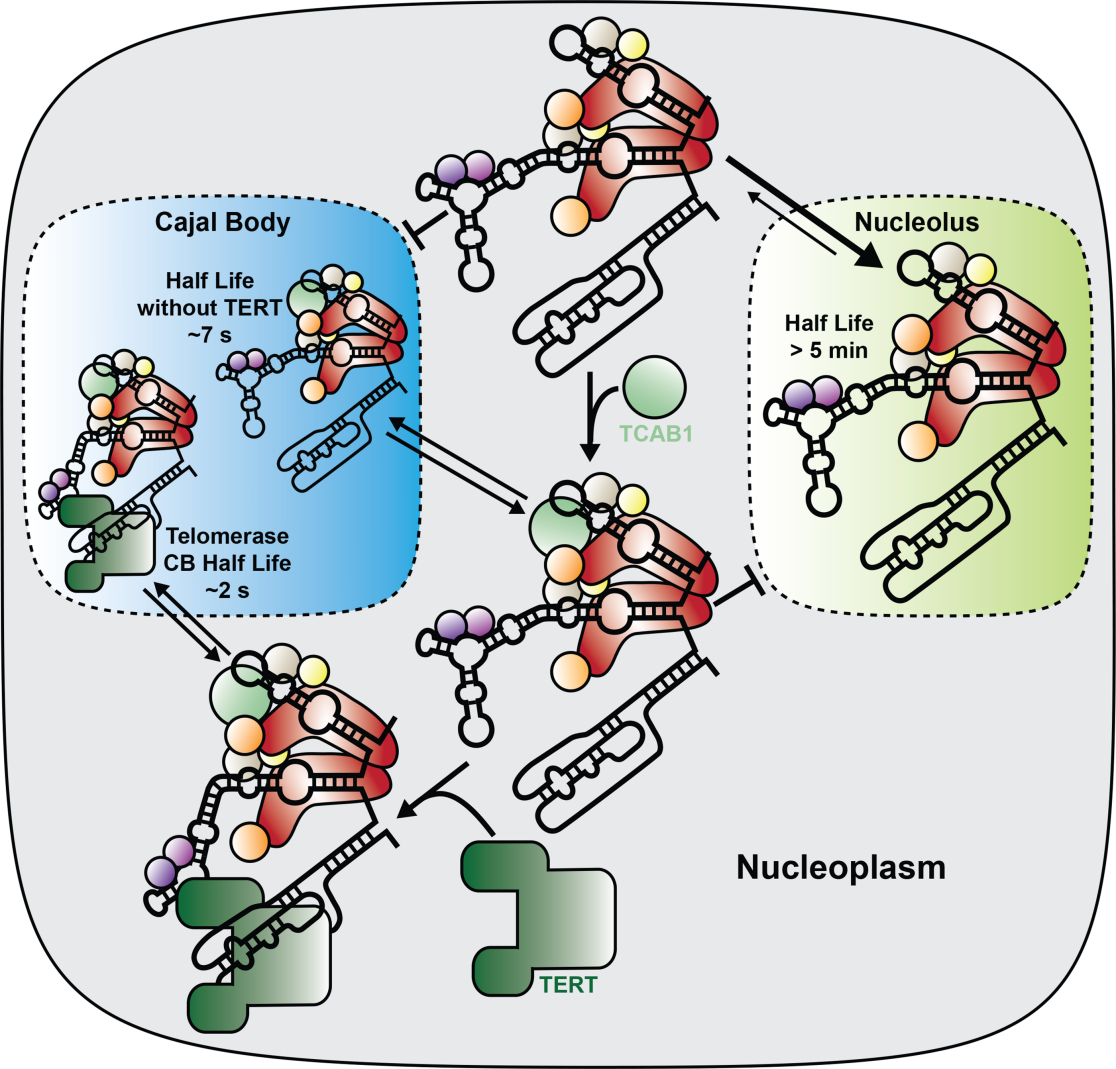
Dynamics of telomerase assembly. The telomerase RNA interacts tightly with the nucleolus in the absence of TCAB1. Once TCAB1 is bound TR entry into the nucleolus is inhibited and it dynamically interacts with Cajal bodies (*t*_1/2_ = 7 s, Laprade et al. [57]). TERT is largely excluded from nucleoli and once telomerase is fully assembled its affinity for Cajal bodies is reduced (*t*_1/2_ = 2 s, Laprade et al. [57]).

The observations described above raise the question: What is the primary function of the H/ACA domain of TR, and why does TR resemble a scaRNA rather than a snoRNA? To address this question Vogan et al. [38] replaced the H/ACA domain of TR with alternative RNA sequences aimed at stabilizing TR. This hTRmin variant was able to maintain telomere length independently of TCAB1 and coilin, but it required TERT overexpression, demonstrating that its assembly with TERT was possible, but potentially inefficient. This result suggests that the primary function of the H/ACA region of TR is to promote its stability and potentially efficient assembly of TR with TERT. To understand why TR is a scaRNA, we have to focus on the role TCAB1 plays in telomerase RNP function. Depletion of TCAB1 leads to TR accumulation in nucleoli, essentially resembling snoRNA (Figure 3) [34]. TERT on the other hand, is largely excluded from nucleoli [39], and has been suggested to contain a nuclear localization signal [54]. The spatial separation of TERT and TR in the absence of TCAB1 reduces, but does not completely prevent, telomerase assembly, which explains why TCAB1 knock-out cells retain proliferative potential but have a short telomere length set point [36,38,39]. Like TERT, TCAB1 does not enter nucleoli, therefore, it likely encounters TR in the nucleoplasm and prevents its entry into nucleoli [39]. As mentioned above, a reduction in TGS1 activity, also leads to the accumulation of TR in nucleoli, however in this case, telomerase activity is increased [25]. This implies that TR accumulation in nucleoli is not always accompanied by a decrease in telomerase activity. It is possible that a reduction in TGS1 activity leads to the accumulation of assembled telomerase in the nucleolus, or alternatively TR could dynamically shuttle in and out of nucleoli when it lacks a TMG cap. In summary, these observations support a model in which the H/ACA domain stabilizes TR, and TCAB1 promotes its assembly with TERT in the nucleoplasm (Figure 3).

The final component of the telomerase RNP is the recently identified H2A/H2B dimer. Due to its abundance in the nucleus, it is likely that the H2A/H2B dimer associates with TR co-transcriptionally or immediately following TR synthesis (Figure 2). Since the H/ACA domain forms the base of the stem that ends in the CR4/5 region which H2A/H2B recognizes, it is possible that CR4/5 also folds co-transcriptionally. The functional relevance of H2A/H2B binding will be challenging to address because genetic perturbation of H2A/H2B is practically impossible and preventing its binding to TR without functionally disrupting the critical CR4/5 region of TR will not be trivial.

## Dynamics of telomerase assembly

The analysis of telomerase biogenesis requires reliable detection of its components. Due to their low expression levels (∼1000 molecules per cell in cancer cells), both TERT and TR are not straightforward to study. Recently, live-cell imaging approaches have been developed to detect both TERT and TR with single-molecule sensitivity in human cells [55–57]. These methods are based on fluorescent tags that were introduced at the endogenous TERT and TR loci, respectively, preserving normal expression levels and regulatory mechanisms conferred by their respective genomic context. The vast majority of both TERT and TR freely diffuse around the nucleus at any given time, they are depleted from nucleoli and both components were shown to interact with CBs, as expected [55,57]. Interestingly, the association of TR with CBs is fairly dynamic when TERT is present in cells (*t*_1/2_ = 2 s) and the residence time of TR in CBs is increased when TERT is knocked out (*t*_1/2_ = 7 s) (Figure 3). This suggests that the affinity of fully assembled telomerase for CBs is lower than that of TR not bound by TERT. The reduced binding of telomerase to CBs could be critical to facilitate telomerase recruitment to telomeres by preventing its retention in CBs. In the absence of TCAB1, the interaction of TR with Cajal bodies is completely absent [57], and a large fraction of TR is tightly associated with the nucleolus, inhibiting it from encountering TERT (Figure 3) [39]. Together these observations support a model in which TERT encounters TR in the nucleoplasm, or in CBs, although their presence is not strictly required (Figure 3).

In summary, our understanding of the molecular principles underlying telomerase assembly has advanced tremendously over the last decade. The structural, cell biological, and biochemical tools are in place to address the remaining key unanswered questions [46,58–60], for instance how 5′- and 3′-end maturation regulates and is coordinated with telomerase RNP assembly. Ultimately, the goal in the field is to define critical intervention points, such as the inhibition of PAPD5 and potentially TGS1 [20,23,61,62], that can be targeted to ameliorate deficiencies in telomere maintenance or to inhibit telomerase activity in cancer cells. Telomerase biogenesis is a complex multi-step process that may provide various avenues to attain this goal.

## Perspectives

- Telomerase is a complex ribonucleoprotein composed of the telomerase RNA and 12 protein co-factors. Telomere maintenance by telomerase is essential for the proliferation of stem cells and germ cells as well as most cancers. Defining the molecular mechanisms of telomerase assembly is therefore critical to understand stem cell biology and potentially develop cancer therapies targeting telomerase activity.
- Telomerase assembly is a multi-step process including co-transcriptional assembly of the H/ACA lobe of telomerase, followed by post-transcriptional modification of the 5′- end 3′-ends of the telomerase RNA, and concluding with the association with the telomerase reverse transcriptase. The telomerase components dynamically associate with Cajal bodies and nucleoli, but the precise order and location of critical telomerase biogenesis steps is still unknown.
- Cryo-EM analysis of telomerase assembly intermediates and live cell imaging of critical telomerase components in combination with structural mapping of the telomerase RNA will provide important insights into telomerase assembly. These findings will potentially be leverage to target telomerase assembly to treat pre-mature ageing diseases and cancer.

## Abbreviations

CAB: Cajal body
CBs: Cajal bodies
Cryo-EM: cryogenic electron microscopy
NHP2: non-Histone Protein 2
NOP10: nucleolar Protein 10
PARN: poly(A)-specific ribonuclease
RNP: ribonucleoprotein
scaRNAs: small Cajal body RNAs
snoRNAs: small nucleolar RNAs
snRNAs: small nuclear RNAs
TCAB1: telomerase Cajal body protein 1
TGS1: trimethyguanosine synthase 1
TMG: trimethyguanosine
TR: telomerase RNA
TRBD: telomerase RNA-binding domain

## References

[1] Moyzis, R.K., Buckingham, J.M., Cram, L.S., Dani, M., Deaven, L.L., Jones, M.D. et al. (1988) A highly conserved repetitive DNA sequence, (TTAGGG)n, present at the telomeres of human chromosomes. Proc. Natl Acad. Sci. U.S.A. 85, 6622–6626 10.1073/pnas.85.18.6622 3413114 PMC282029

[2] Harley, C.B., Futcher, A.B. and Greider, C.W. (1990) Telomeres shorten during ageing of human fibroblasts. Nature 345, 458–460 10.1038/345458a0 2342578

[3] Egan, E.D. and Collins, K. (2010) Specificity and stoichiometry of subunit interactions in the human telomerase holoenzyme assembled *in vivo*. Mol. Cell. Biol. 30, 2775–2786 10.1128/mcb.00151-10 20351177 PMC2876521

[4] Ghanim, G.E., Fountain, A.J., van Roon, A.-M.M., Rangan, R., Das, R., Collins, K. et al. (2021) Structure of human telomerase holoenzyme with bound telomeric DNA. Nature 593, 449–453 10.1038/s41586-021-03415-4 33883742 PMC7610991

[5] Nguyen, T.H.D., Tam, J., Wu, R.A., Greber, B.J., Toso, D., Nogales, E. et al. (2018) Cryo-EM structure of substrate-bound human telomerase holoenzyme. Nature 557, 190–195 10.1038/s41586-018-0062-x 29695869 PMC6223129

[6] Wan, F., Ding, Y., Zhang, Y., Wu, Z., Li, S., Yang, L. et al. (2021) Zipper head mechanism of telomere synthesis by human telomerase. Cell Res. 31, 1275–1290 10.1038/s41422-021-00586-7 34782750 PMC8648750

[7] Liu, B., He, Y., Wang, Y., Song, H., Zhou, Z.H. and Feigon, J. (2022) Structure of active human telomerase with telomere shelterin protein TPP1. Nature 604, 578–583 10.1038/s41586-022-04582-8 35418675 PMC9912816

[8] Armanios, M. and Blackburn, E.H. (2012) The telomere syndromes. Nat. Rev. Genet. 13, 693–704 10.1038/nrg3246 22965356 PMC3548426

[9] Glousker, G., Touzot, F., Revy, P., Tzfati, Y. and Savage, S.A. (2015) Unraveling the pathogenesis of Hoyeraal–Hreidarsson syndrome, a complex telomere biology disorder. Br. J. Haematol. 170, 457–471 10.1111/bjh.13442 25940403 PMC4526362

[10] Kim, N.W., Piatyszek, M.A., Prowse, K.R., Harley, C.B., West, M.D., Ho, P.L.C. et al. (1994) Specific association of human telomerase activity with immortal cells and cancer. Science 266, 2011–2015 10.1126/science.7605428 7605428

[11] Feng, J., Funk, W., Wang, S., Weinrich, S., Avilion, A., Chiu, C. et al. (1995) The RNA component of human telomerase. Science 269, 1236–1241 10.1126/science.7544491 7544491

[12] Fu, D. and Collins, K. (2003) Distinct biogenesis pathways for human telomerase RNA and H/ACA small nucleolar RNAs. Mol. Cell 11, 1361–1372 10.1016/s1097-2765(03)00196-5 12769858

[13] Jády, B.E., Bertrand, E. and Kiss, T. (2004) Human telomerase RNA and box H/ACA scaRNAs share a common Cajal body–specific localization signal. J. Cell Biol. 164, 647–652 10.1083/jcb.200310138 14981093 PMC2172171

[14] Darzacq, X., Kittur, N., Roy, S., Shav-Tal, Y., Singer, R.H. and Meier, U.T. (2006) Stepwise RNP assembly at the site of H/ACA RNA transcription in human cells. J. Cell Biol. 173, 207–218 10.1083/jcb.200601105 16618814 PMC2063812

[15] Tseng, C.-K., Wang, H.-F., Burns, A.M., Schroeder, M.R., Gaspari, M. and Baumann, P. (2015) Human telomerase RNA processing and quality control. Cell Rep. 13, 2232–2243 10.1016/j.celrep.2015.10.075 26628367

[16] Nguyen, D., Grenier St-Sauveur, V., Bergeron, D., Dupuis-Sandoval, F., Scott, M.S. and Bachand, F. (2015) A polyadenylation-dependent 3′ end maturation pathway is required for the synthesis of the human telomerase RNA. Cell Rep. 13, 2244–2257 10.1016/j.celrep.2015.11.003 26628368

[17] Moon, D.H., Segal, M., Boyraz, B., Guinan, E., Hofmann, I., Cahan, P. et al. (2015) Poly(A)-specific ribonuclease (PARN) mediates 3′-end maturation of the telomerase RNA component. Nat. Genet. 47, 1482–1488 10.1038/ng.3423 26482878 PMC4791094

[18] Tseng, C.-K., Wang, H.-F., Schroeder, M.R. and Baumann, P. (2018) The H/ACA complex disrupts triplex in hTR precursor to permit processing by RRP6 and PARN. Nat. Commun. 9, 5430 10.1038/s41467-018-07822-6 30575725 PMC6303318

[19] Shukla, S., Schmidt, J.C., Goldfarb, K.C., Cech, T.R. and Parker, R. (2016) Inhibition of telomerase RNA decay rescues telomerase deficiency caused by dyskerin or PARN defects. Nat. Struct. Mol. Biol. 23, 286–292 10.1038/nsmb.3184 26950371 PMC4830462

[20] Shukla, S., Jeong, H.-C., Sturgeon, C.M., Parker, R. and Batista, L.F.Z. (2020) Chemical inhibition of PAPD5/7 rescues telomerase function and hematopoiesis in dyskeratosis congenita. Blood Adv. 4, 2717–2722 10.1182/bloodadvances.2020001848 32559291 PMC7322949

[21] Boyraz, B., Moon, D.H., Segal, M., Muosieyiri, M.Z., Aykanat, A., Tai, A.K. et al. (2016) Posttranscriptional manipulation of TERC reverses molecular hallmarks of telomere disease. J. Clin. Invest. 126, 3377–3382 10.1172/jci87547 27482890 PMC5004950

[22] Mitchell, J.R., Wood, E. and Collins, K. (1999) A telomerase component is defective in the human disease dyskeratosis congenita. Nature 402, 551–555 10.1038/990141 10591218

[23] Nagpal, N., Wang, J., Zeng, J., Lo, E., Moon, D.H., Luk, K. et al. (2020) Small-molecule PAPD5 inhibitors restore telomerase activity in patient stem cells. Cell Stem Cell 26, 896–909.e8 10.1016/j.stem.2020.03.016 32320679 PMC7275922

[24] Verheggen, C. and Bertrand, E. (2012) CRM1 plays a nuclear role in transporting snoRNPs to nucleoli in higher eukaryotes. Nucleus 3, 132–137 10.4161/nucl.19266 22555597 PMC3383567

[25] Chen, L., Roake, C.M., Galati, A., Bavasso, F., Micheli, E., Saggio, I. et al. (2020) Loss of human TGS1 hypermethylase promotes increased telomerase RNA and telomere elongation. Cell Rep. 30, 1358–1372.e5 10.1016/j.celrep.2020.01.004 32023455 PMC7156301

[26] Buemi, V., Schillaci, O., Santorsola, M., Bonazza, D., Broccia, P.V., Zappone, A. et al. (2022) TGS1 mediates 2,2,7-trimethyl guanosine capping of the human telomerase RNA to direct telomerase dependent telomere maintenance. Nat. Commun. 13, 2302 10.1038/s41467-022-29907-z 35484160 PMC9050681

[27] Schlotter, F., Mérouani, S., Flayac, J., Kogey, V., Issa, A., Dodré, M. et al. (2023) Proteomic analyses reveal new features of the box H/ACA RNP biogenesis. Nucleic Acids Res. 51, 3357–3374 10.1093/nar/gkad129 36869663 PMC10123114

[28] MacNeil, D.E., Lambert-Lanteigne, P., Qin, J., McManus, F.P., Bonneil, E., Thibault, P. et al. (2021) SUMOylation- and GAR1-dependent regulation of dyskerin nuclear and subnuclear localization. Mol. Cell. Biol. 41, e00464-20 10.1128/mcb.00464-20 33526451 PMC8088130

[29] Grozdanov, P.N., Roy, S., Kittur, N. and Meier, U.T. (2009) SHQ1 is required prior to NAF1 for assembly of H/ACA small nucleolar and telomerase RNPs. RNA 15, 1188–1197 10.1261/rna.1532109 19383767 PMC2685518

[30] Walbott, H., Machado-Pinilla, R., Liger, D., Blaud, M., Réty, S., Grozdanov, P.N. et al. (2011) The H/ACA RNP assembly factor SHQ1 functions as an RNA mimic. Genes Dev. 25, 2398–2408 10.1101/gad.176834.111 22085966 PMC3222905

[31] Machado-Pinilla, R., Liger, D., Leulliot, N. and Meier, U.T. (2012) Mechanism of the AAA+ ATPases pontin and reptin in the biogenesis of H/ACA RNPs. RNA 18, 1833–1845 10.1261/rna.034942.112 22923768 PMC3446707

[32] Fatica, A., Dlakić, M. and Tollervey, D. (2002) Naf1 p is a box H/ACA snoRNP assembly factor. RNA (New York, N.Y.) 8, 1502–1514 10.1017/S1355838202022094 12515383 PMC1370356

[33] Stanley, S.E., Gable, D.L., Wagner, C.L., Carlile, T.M., Hanumanthu, V.S., Podlevsky, J.D. et al. (2016) Loss-of-function mutations in the RNA biogenesis factor NAF1 predispose to pulmonary fibrosis–emphysema. Sci. Transl. Med. 8, 351ra107 10.1126/scitranslmed.aaf7837 PMC5351811 27510903

[34] Venteicher, A.S., Abreu, E.B., Meng, Z., McCann, K.E., Terns, R.M., Veenstra, T.D. et al. (2009) A human telomerase holoenzyme protein required for Cajal body localization and telomere synthesis. Science 323, 644–648 10.1126/science.1165357 19179534 PMC2728071

[35] Zhong, F., Savage, S.A., Shkreli, M., Giri, N., Jessop, L., Myers, T. et al. (2011) Disruption of telomerase trafficking by TCAB1 mutation causes dyskeratosis congenita. Gene Dev 25, 11–16 10.1101/gad.2006411 21205863 PMC3012932

[36] Chen, L., Roake, C.M., Freund, A., Batista, P.J., Tian, S., Yin, Y.A. et al. (2018) An activity switch in human telomerase based on RNA conformation and shaped by TCAB1. Cell 174, 218–230.e13 10.1016/j.cell.2018.04.039 29804836 PMC6063371

[37] Freund, A., Zhong, F.L., Venteicher, A.S., Meng, Z., Veenstra, T.D., Frydman, J. et al. (2014) Proteostatic control of telomerase function through TRiC-mediated folding of TCAB1. Cell 159, 1389–1403 10.1016/j.cell.2014.10.059 25467444 PMC4329143

[38] Vogan, J.M., Zhang, X., Youmans, D.T., Regalado, S.G., Johnson, J.Z., Hockemeyer, D. et al. (2016) Minimized human telomerase maintains telomeres and resolves endogenous roles of H/ACA proteins, TCAB1, and Cajal bodies. eLife 5, e18221 10.7554/elife.18221 27525486 PMC5005035

[39] Klump, B.M., Perez, G.I., Patrick, E.M., Adams-Boone, K., Cohen, S.B., Han, L. et al. (2023) TCAB1 prevents nucleolar accumulation of the telomerase RNA to facilitate telomerase assembly. Cell Rep. 42, 112577 10.1016/j.celrep.2023.112577 37267110 PMC10569210

[40] Bergstrand, S., Böhm, S., Malmgren, H., Norberg, A., Sundin, M., Nordgren, A. et al. (2020) Biallelic mutations in WRAP53 result in dysfunctional telomeres, Cajal bodies and DNA repair, thereby causing Hoyeraal–Hreidarsson syndrome. Cell Death Dis. 11, 238 10.1038/s41419-020-2421-4 32303682 PMC7165179

[41] Ueda, Y., Calado, R.T., Norberg, A., Kajigaya, S., Roos, G., Hellstrom-Lindberg, E. et al. (2014) A mutation in the H/ACA box of telomerase RNA component gene (TERC) in a young patient with myelodysplastic syndrome. BMC Med. Genet. 15, 68–68 10.1186/1471-2350-15-68 24948335 PMC4073180

[42] Stern, J.L., Zyner, K.G., Pickett, H.A., Cohen, S.B. and Bryan, T.M. (2012) Telomerase recruitment requires both TCAB1 and Cajal bodies independently. Mol. Cell. Biol. 32, 2384–2395 10.1128/mcb.00379-12 22547674 PMC3434490

[43] Theimer, C.A., Blois, C.A. and Feigon, J. (2005) Structure of the human telomerase RNA pseudoknot reveals conserved tertiary interactions essential for function. Mol. Cell 17, 671–682 10.1016/j.molcel.2005.01.017 15749017

[44] Chen, J.-L. and Greider, C.W. (2005) Functional analysis of the pseudoknot structure in human telomerase RNA. Proc. Natl Acad. Sci. U.S.A. 102, 8080–8085 10.1073/pnas.0502259102 15849264 PMC1149427

[45] Lai, C.K., Mitchell, J.R. and Collins, K. (2001) RNA binding domain of telomerase reverse transcriptase. Mol. Cell. Biol. 21, 990–1000 10.1128/mcb.21.4.990-1000.2001 11158287 PMC99554

[46] Forino, N.M., Woo, J.Z., Zaug, A.J., Jimenez, A.G., Cech, T.R., Rouskin, S. et al. (2023) Dissecting telomerase RNA structural heterogeneity in living human cells with DMS-MaPseq. bioRxiv 10.1101/2023.10.04.560962 PMC11754830 39843442

[47] Xi, L. and Cech, T.R. (2014) Inventory of telomerase components in human cells reveals multiple subpopulations of hTR and hTERT. Nucleic Acids Res. 42, 8565–8577 10.1093/nar/gku560 24990373 PMC4117779

[48] Chiba, K., Johnson, J.Z., Vogan, J.M., Wagner, T., Boyle, J.M. and Hockemeyer, D. (2015) Cancer-associated TERT promoter mutations abrogate telomerase silencing. eLife 4, e07918 10.7554/elife.07918 26194807 PMC4507476

[49] Wang, Q., Sawyer, I.A., Sung, M.-H., Sturgill, D., Shevtsov, S.P., Pegoraro, G. et al. (2016) Cajal bodies are linked to genome conformation. Nat. Commun. 7, 10966 10.1038/ncomms10966 26997247 PMC4802181

[50] Gallardo, F., Olivier, C., Dandjinou, A.T., Wellinger, R.J. and Chartrand, P. (2008) TLC1 RNA nucleo-cytoplasmic trafficking links telomerase biogenesis to its recruitment to telomeres. EMBO J. 27, 748–757 10.1038/emboj.2008.21 18273059 PMC2265757

[51] Lubas, M., Christensen, M.S., Kristiansen, M.S., Domanski, M., Falkenby, L.G., Lykke-Andersen, S. et al. (2011) Interaction profiling identifies the human nuclear exosome targeting complex. Mol. Cell 43, 624–637 10.1016/j.molcel.2011.06.028 21855801

[52] Berndt, H., Harnisch, C., Rammelt, C., Stöhr, N., Zirkel, A., Dohm, J.C. et al. (2012) Maturation of mammalian H/ACA box snoRNAs: PAPD5-dependent adenylation and PARN-dependent trimming. RNA 18, 958–972 10.1261/rna.032292.112 22442037 PMC3334704

[53] Chen, Y., Deng, Z., Jiang, S., Hu, Q., Liu, H., Songyang, Z. et al. (2015) Human cells lacking coilin and Cajal bodies are proficient in telomerase assembly, trafficking and telomere maintenance. Nucleic Acids Res. 43, 385–395 10.1093/nar/gku1277 25477378 PMC4288172

[54] Chung, J., Khadka, P. and Chung, I.K. (2012) Nuclear import of hTERT requires a bipartite nuclear localization signal and Akt-mediated phosphorylation. J. Cell Sci. 125, 2684–2697 10.1242/jcs.099267 22366458

[55] Schmidt, J.C., Zaug, A.J. and Cech, T.R. (2016) Live cell imaging reveals the dynamics of telomerase recruitment to telomeres. Cell 166, 1188–1197.e9 10.1016/j.cell.2016.07.033 27523609 PMC5743434

[56] Schmidt, J.C., Zaug, A.J., Kufer, R. and Cech, T.R. (2018) Dynamics of human telomerase recruitment depend on template-telomere base pairing. Mol. Biol. Cell 29, 869–880 10.1091/mbc.e17-11-0637 29386295 PMC5905299

[57] Laprade, H., Querido, E., Smith, M.J., Guérit, D., Crimmins, H., Conomos, D. et al. (2020) Single-molecule imaging of telomerase RNA reveals a recruitment-retention model for telomere elongation. Mol. Cell 79, 115–126.e6 10.1016/j.molcel.2020.05.005 32497497

[58] Chen, L., Chang, H.Y. and Artandi, S.E. (2021) Analysis of RNA conformation in endogenously assembled RNPs by icSHAPE. STAR Protoc. 2, 100477 10.1016/j.xpro.2021.100477 33997809 PMC8102169

[59] Palka, C., Forino, N., Hentschel, J., Das, R. and Stone, M.D. (2020) Folding heterogeneity in the essential human telomerase RNA three-way junction. RNA 26, 1787–1800 10.1261/rna.077255.120 32817241 PMC7668248

[60] Niederer, R.O. and Zappulla, D.C. (2015) Refined secondary-structure models of the core of yeast and human telomerase RNAs directed by SHAPE. RNA 21, 254–261 10.1261/rna.048959.114 25512567 PMC4338352

[61] Chu, C.-M., Yu, H.-H., Kao, T.-L., Chen, Y.-H., Lu, H.-H., Wu, E.-T. et al. (2022) A missense variant in the nuclear localization signal of DKC1 causes Hoyeraal-Hreidarsson syndrome. NPJ Genom. Med. 7, 64 10.1038/s41525-022-00335-8 36309505 PMC9617742

[62] Galati, A., Scatolini, L., Micheli, E., Bavasso, F., Cicconi, A., Maccallini, P. et al. (2022) The S-adenosylmethionine analog sinefungin inhibits the trimethylguanosine synthase TGS1 to promote telomerase activity and telomere lengthening. FEBS Lett. 596, 42–52 10.1002/1873-3468.14240 34817067

